# Factors associated with antegrade true-sub-true phenomenon in percutaneous coronary intervention for chronic total occlusion

**DOI:** 10.1371/journal.pone.0232158

**Published:** 2020-04-24

**Authors:** Kei Yamamoto, Kenichi Sakakura, Takunori Tsukui, Masaru Seguchi, Yousuke Taniguchi, Hiroshi Wada, Shin-ichi Momomura, Hideo Fujita

**Affiliations:** Division of Cardiovascular Medicine, Saitama Medical Center, Jichi Medical University, Saitama, Japan; Antonius Ziekenhuis, NETHERLANDS

## Abstract

**Background:**

Recently, the importance of chronic total occlusion (CTO)-percutaneous coronary intervention (PCI) has been emphasized with greater success rates. In the antegrade wire based approach, it is generally considered that the guidewire would not advance from the subintimal space to the intimal space without dissection re-entry device. However, it is sometimes observed by intravascular ultrasound (IVUS) that the guidewire within the subintimal space advanced into the distal true lumen. The purpose of this study was to investigate specific conditions or characteristics which were associated with “antegrade true-sub-true” phenomenon in CTO-PCI.

**Methods:**

We retrospectively reviewed consecutive 320 CTO lesions that underwent CTO-PCI in our institution. Among them, 16 lesions in which the IVUS confirmed the “antegrade true-sub-true” phenomenon were categorized as the true-sub-true group, whereas 27 lesions that resulted in unsuccessful CTO-PCI were categorized as the unsuccessful group. We compared the clinical, lesion, and procedural characteristics between the true-sub-true group and the unsuccessful group.

**Results:**

The prevalence of bifurcation with abrupt type in CTO exit-sites was significantly higher in the true-sub-true group in comparison to the unsuccessful group (75.0% vs. 25.9%, p = 0.002). The multivariate logistic regression analysis revealed that bifurcation with abrupt type in CTO exit-site (OR 8.017; 95%CI: 1.484–43.304; p = 0.016) was independent predictor of the antegrade true-sub-true phenomenon.

**Conclusions:**

In CTO-PCI, the antegrade true-sub-true phenomenon is rare, but can be a last chance for successful PCI. Bifurcation with abrupt type in CTO exit-site was significantly associated with the antegrade true-sub-true phenomenon.

## Introduction

Although the benefit of percutaneous coronary intervention (PCI) to chronic total occlusion (CTO) has not been established, PCI to CTO may be associated with the improvement of cardiac function and clinical outcomes [1–3]. Recent advancements of interventional devices and techniques have achieved the greater success rate and favorable outcomes [1, 4–6]. Of note, reverse controlled antegrade-retrograde tracking (CART) was a novel technique, which increased the success rate of CTO-PCI up to 80–90% [7–9]. Despite of these advancements, unsuccessful procedures are still observed in the contemporary CTO-PCI, especially when interventional collaterals are not available [10]. If interventional collaterals are not present, the choice might be the antegrade wire based approach [11].

In the antegrade wire based approach, if the guidewire advanced into the subintimal space, subintimal tracking and reentry (STAR) technique [12], dissection re-entry device (Stingray System™) or parallel wiring could be the choice [11, 13, 14]. Furthermore, if both dissection re-entry device and parallel wiring failed, intravascular ultrasound (IVUS)-guided wiring might be the last resort [11]. A key to success in IVUS-guided wiring is to identify the entry point of wire entering from the true lumen to the false lumen, and to pullback the guidewire until that entry point [15], because it is generally considered that the guidewire would not advance from the subintimal space to the intimal space without dissection re-entry device [16, 17]. Moreover, from the pathologic view, it would be rare for the guidewire within the subintimal space to advance into the intimal true lumen, because the occluded intimal plaques, which are mainly composed of type I collagen [18], would be harder than the subintimal space, especially when the subintimal space was expanded [19]. Nevertheless, it is sometimes observed by IVUS that the guidewire within the subintimal space advanced into the distal true lumen without dissection re-entry devices/retrograde approach, which can be called as “antegrade true-sub-true” phenomenon. We hypothesized that this “antegrade true-sub-true” phenomenon would not happen accidentally, but could happen under some specific conditions. If we can recognize such specific conditions, the success rate of CTO-PCI may further increase. The purpose of this study was to investigate specific conditions or characteristics which were associated with “antegrade true-sub-true” phenomenon in CTO-PCI.

## Methods

### Study design

The present study was a retrospective and single center study. We reviewed 320 patients who underwent PCI for CTO lesions between January 2014 and June 2019. CTO lesions were defined as 100% coronary occlusion with Thrombolysis in Myocardial Infarction grade 0 distal flow persistent for >3 months. After the guidewire successfully crossed the CTO lesions, we performed IVUS following small balloon dilatation to confirm whether the guidewire was within true lumen or subintimal space in all the cases. The inclusion criterion was CTO lesions that we attempted to revascularize by PCI during the study period. The exclusion criteria were (1) successful PCI cases in which the guidewire was crossed through intraplaque (all true lumen), which were confirmed by IVUS, (2) successful PCI cases in which the guidewire was finally crossed by retrograde techniques (mainly reverse CART). After inclusion and exclusion criteria were applied, unsuccessful CTO lesions were included as the unsuccessful group. Successful CTO lesions in which the guidewire was crossed by the antegrade wiring through subintimal space identified by IVUS were included as the true-sub-true group. We compared the clinical, lesion, and procedural characteristics between the unsuccessful group and the true-sub-true group. This study was approved by the institutional review board of Saitama Medical Center, Jichi Medical University, and written informed consent was waived, because of the retrospective study design.

### Definitions

**Fig 1:**
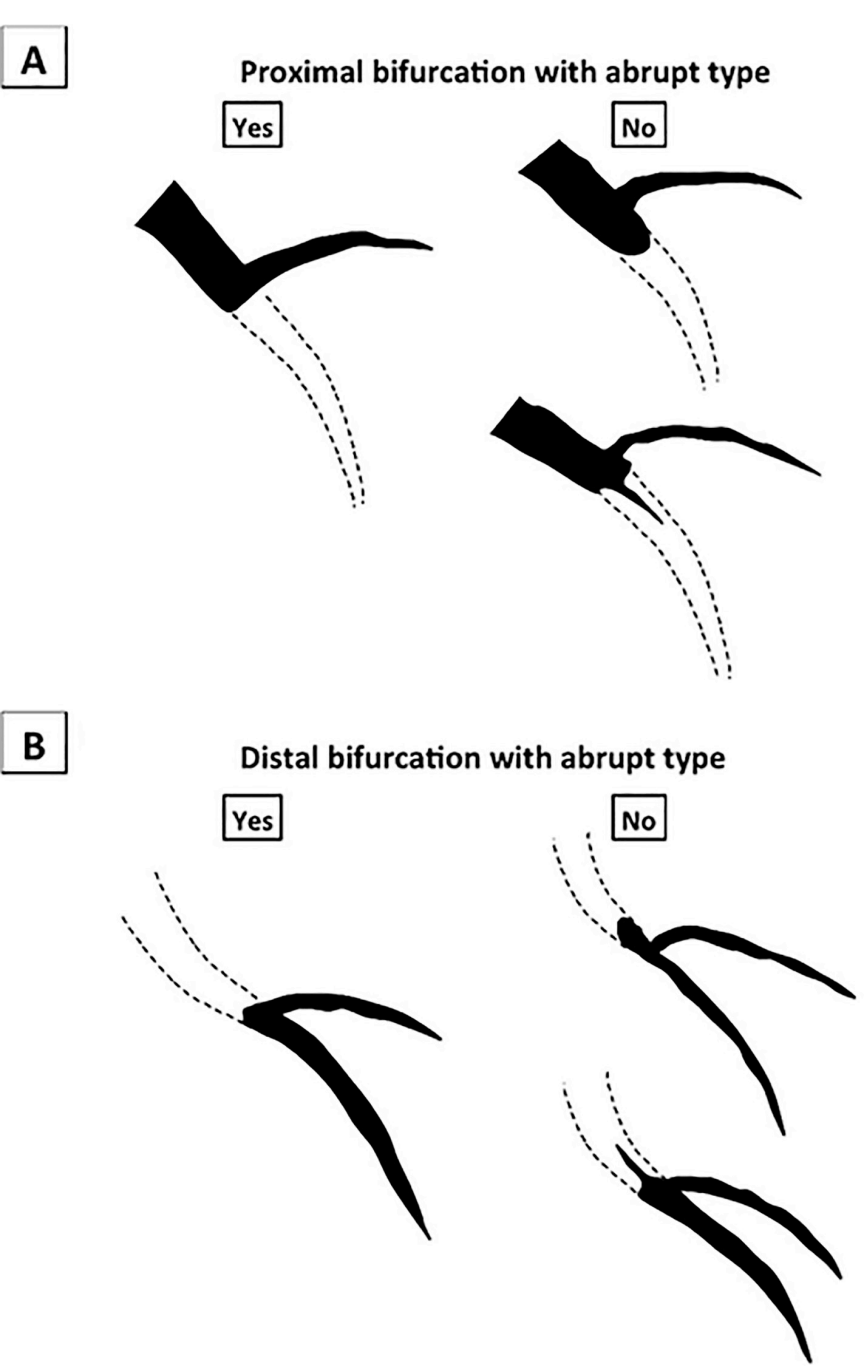
The schema of bifurcation with abrupt type.

The body surface area (BSA) was calculated with the DuBois formula, as follows: BSA = [body weight (kg)]^0.425^×[body height (cm)]^0.725^×0.007184 [20]. Hypertension was defined as systolic blood pressure >140 mmHg, diastolic blood pressure >90 mmHg, or medical treatment for hypertension [21]. Diabetes mellitus was defined as a hemoglobin A1c level >6.5% or treatment for diabetes mellitus [22]. Hyperlipidemia was defined as a total cholesterol level >220 mg/dl, a low-density lipoprotein cholesterol level >140 mg/dl, or treatment for hyperlipidemia [22]. Chronic kidney disease was defined as estimated glomerular filtration rate <60 ml/min at admission [22]. Collateral flow was evaluated by Rentrop classification [23]. Abrupt type was defined as the occluded segment that did not end in a funnel-shaped form, which was the same as the blunt type in the J-CTO score [24]. Bifurcation with abrupt type was defined as the abrupt type that had a branch at the occluded segment (Fig 1). Mid-island was defined as a spot area that was filled with contrast media within the CTO lesion. The J-CTO score was calculated as previously described [25]. In brief, the J-CTO score includes 5 morphologic characteristics of a CTO: blunt proximal cap, calcification, bending >45°, length of occluded segment >20 mm, and re-attempt [25]. Presence of calcification was evaluated by angiography [26]. Bending was defined as at least 1 bend of >45° assessed by angiography within CTO [25]. In clinical practice, J-CTO score would be calculated with an estimation of the occlusion length. However, we retrospectively calculated the J-CTO score for the present study, and adopted the occlusion length measured by quantitative coronary angiography (QCA). QCA parameters were measured using a cardiovascular angiography analysis system (QAngio XA 7.3, MEDIS Imaging Systems, Leiden, Netherlands). Since it was impossible to draw the line for QCA in total occlusion, the occlusion length and reference diameter were measured after small balloon dilatation. Antegrade sub-true was defined referring IVUS image. The “antegrade true-sub-true” phenomenon, which was the requisite condition for the true-sub-true group, was retrospectively confirmed by IVUS images after guidewire crossing. The “antegrade true-sub-true” phenomenon was not derived from intentional re-entry procedures in the IVUS-guide wiring, because we tried to advance the guidewire through intraplaque and pulled back the guidewire when the guidewire was identified in the subintimal space in the IVUS-guide wiring.

### Percutaneous coronary intervention of the chronic total occlusion

The CTO-PCI were performed using standard techniques via radial artery, femoral artery. A 7-Fr or 8-Fr system was preferred for CTO-PCI. In transradial approach, we used glide-sheath 7Fr (Terumo, Japan), conventional 7Fr sheath (Medikit, Japan), or 6Fr sheath (Medikit, Japan). We usually inserted sheath into contralateral artery to confirm collateral flow or to perform retrograde PCI. In this study population, bidirectional approach were performed in 86% of study cases (16 bi-femoral approach, 21 radial and femoral approach). We also keep the venous route using 5-Fr sheath to check activated coagulation time, which was maintained >300 seconds during PCI. Generally, we discussed CTO strategy before procedures referring angiographic information such as parameters of J-CTO score, renal function, or collateral flow grade [23]. If the occlusion length was short with tapered entry, we started the procedure with the standard antegrade penetration. The parallel wire technique was considered if an antegrade wire went into the subintimal space, but the distal lumen was still visible [27]. Antegrade dissection re-entry devices were not available in our institution during the study period. Bidirectional approach was considered if the occlusion length was long with abrupt entry and the presence of interventional collateral was confirmed [8]. An IVUS guide technique was considered if we could not re-direct the wire from subintimal space to the intraplaque after using other techniques [28]. We considered the termination of procedure in the case that we could not progress PCI using the option of the above procedures, fluoroscopic time or contrast media wasted much over, or could not continue because of complications. We consider that the maximum peak skin dose was approximately 5 Gray and maximum allowable contrast dose (MACD) was approximately 300 ml, however these decision of termination was depend on operators. Most procedures during the study period were performed by 2 operators (K. Yamamoto, Y. Taniguchi) and 1 senior operator (K. Sakakura).

### Angiographic evaluation of the chronic total occlusion

The detailed evaluation of CTO was performed using the angiograms that were acquired before revascularization. The length of occluded segment was calculated by quantitative coronary angiography (QCA).

### Intravascular ultrasound

Intravascular ultrasound (IVUS) was used to confirm the true lumen after the guidewire cross following small balloon dilatation, to evaluate the CTO entry, or to perform the IVUS guide wiring technique. We did not try to advance the IVUS catheter into the subintimal space except IVUS-guide wring technique, because such procedure would expand the subintimal space. We preferred Navifocus WR (Terumo, Tokyo, Japan), however sometimes used Eagle eye (Phillips, Tokyo, Japan). Automatic pull-back system was not selected.

### Statistical analysis

Continuous variables are expressed as mean±SD and categorical variables were presented as count with percentages. Categorical variables were compared using the Fisher exact test for 2 by 2 comparisons and the chi square test for 2 by ≥3 comparisons. The Kolmogorov-Smirnov test was performed to determine if the continuous variables were normally distributed. Normally distributed continuous variables were compared between the groups using an unpaired Student *t* test. Otherwise, continuous variables were compared using a Mann-Whitney *U* test. Univariate logistic regression analysis was used to evaluate the relation between various clinical, lesion and procedural parameters and “antegrade true-sub-true”phenomenon. To select covariates independently associated with “antegrade true-sub-true”phenomenon, significant univariate predictors were reassessed by multivariate logistic regression analysis with values for inclusion and elimination set at p≤0.05. All variables were simultaneously adjusted in one step. Statistical analyses were performed using SPSS 24.0/Windows (Chicago, Illinois, USA).

## Results

**Fig 2:**
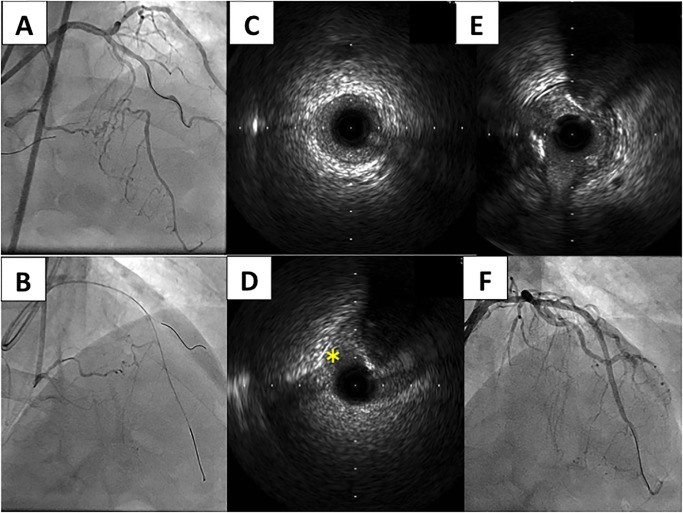
Angiogram and intravascular ultrasound findings of one case in the antegrade true sub-true group. Panel A: Bidirectional coronary angiogram before PCI. Panel B: A stiff guidewire (Conquest Pro 8–20) successfully crossed by antegrade IVUS guide wiring. Panel C: An IVUS image at CTO distal revealed that the guidewire was within the true lumen. Panel D: An IVUS image at CTO revealed that the guidewire was in subintimal space (*). Panel E: An IVUS image at CTO proximal revealed that the guidewire was within the true lumen. Panel F: Final angiogram.

**Fig 3:**
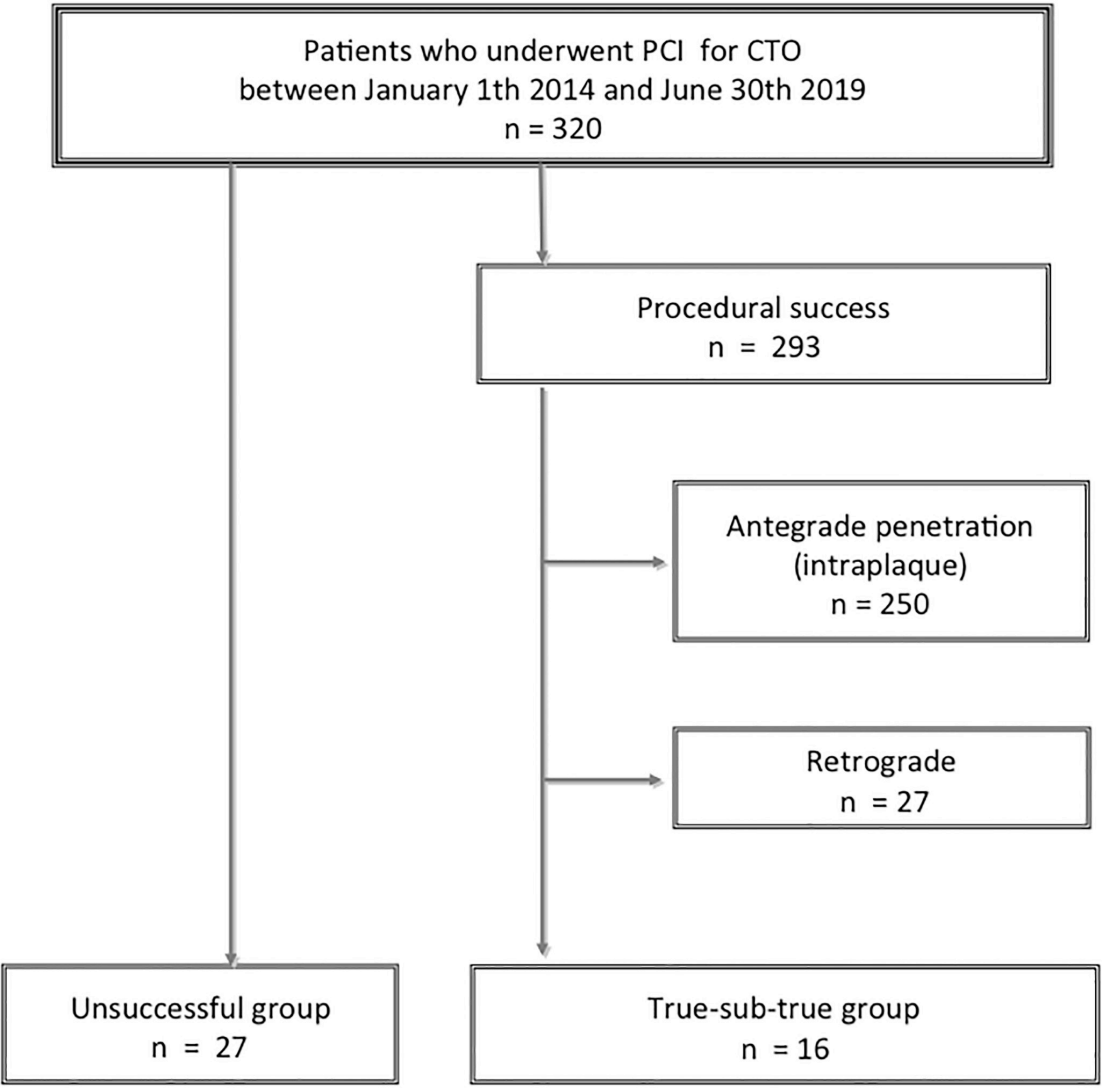
The study flowchart. Abbreviations: PCI, percutaneous coronary intervention; CTO, chronic total occlusion.

Between January 2014 and June 2019, a total of 320 CTO lesions underwent PCI. The overall success rate of CTO-PCI was 91.6% during the study period (85.7% in 2014, 89.3% in 2015, 93.8% in 2017, 94.2% in 2018, and 90.9% in 2019). Among them, 27 lesions resulted in unsuccessful CTO-PCI, and were included as the unsuccessful group. Of 293 successful CTO-PCI lesions, 250 lesions were excluded because the IVUS confirmed that the guidewire was crossed through intraplaque. Furthermore, 27 lesions were excluded, because retrograde approach was applied. Among those 27 lesions, “retrograde true-sub-true” phenomenon was observed in 12 cases (44.4%). Finally, 16 lesions in which the IVUS confirmed the “antegrade true-sub-true” phenomenon were included as the true-sub-true group. A representative case in the true-sub-true group is shown in Fig 2. The study flow chart is shown in Fig 3.

The comparison of clinical characteristics between the 2 groups are shown in Table 1. The clinical characteristics were comparable except gender between the 2 groups. The comparison of lesion and procedural characteristics are shown in Table 2. The prevalence of abrupt type in CTO entry-sites was higher in the unsuccessful group (92.6%) than the true-sub-true group (68.8%) without reaching statistical significance (p = 0.055). The prevalence of bifurcation with abrupt type in CTO exit-sites was significantly higher in the true-sub-true group in comparison to the unsuccessful group (75.0% vs. 25.9%, p = 0.002). Among 12 lesions in which bifurcation with abrupt type in CTO exit-sites, the distal side branch was occluded following revascularization in 5 cases (41.7%). The prevalence of RCA-CTO was less in the true-sub-true group than in the unsuccessful group, whereas the prevalence of LAD-CTO or LCX-CTO was greater in the true-sub-true group than in the unsuccessful group (p = 0.001). There was a trend toward higher J-CTO score in the unsuccessful group as compared to the true-sub-true group (2.67±1.24 vs. 1.94±0.85, p = 0.082). The used devices such as guidewires and microcatheters were comparable between the 2 groups. In the true-sub-true group, the IVUS guide wiring was attempted in 4 patients, while operators tried to advance the guidewire through intimal plaque.

**Table 1:** Comparison of clinical characteristics between the true-sub-true and the PCI unsuccessful groups.

Variables	All (n = 43)	True-sub-true group (n = 16)	Unsuccessful group (n = 27)	p-value
Patient characteristics				
Age ±SD (years)	69±11	69±12	69±10	0.970
Female, n (%)	6 (14.0)	0	6 (22.2)	0.049
Height ±SD (cm)	163.3±8.1	165.3±7.6	162.1±8.3	0.215
Body weight ±SD (kg)	64.3±12.3	66.1±12.6	63.3±12.3	0.303
Body mass index ±SD (kg/m^2^)	24.2±3.4	24.5±3.8	24.0±3.3	0.733
Body surface area ±SD (m^2^)	1.7±0.2	1.8±0.2	1.7±0.2	0.248
Hypertension, n (%)	43 (100)	16 (100)	27 (100)	1.000
Diabetes mellitus, n (%)	13 (30.2)	3 (18.8)	10 (37.0)	0.180
Hyperlipidemia, n (%)	43 (100)	16 (100)	27 (100)	1.000
Chronic kidney disease, n (%)	19 (44.2)	6 (37.5)	13 (48.1)	0.360
Hemodialysis, n (%)	3 (0.237)	0	3 (11.1)	0.237
CT angiography before CTO-PCI, n (%)	29 (67.4)	11 (68.8)	18 (67.7)	0.581

Data are expressed as the mean±SD or number (percentage). A Student’s *t* test was used for normally distributed continuous variables, a Mann-Whitney *U* test was used for abnormally distributed continuous variables, and a Fisher exact test was used for categorical variables. CT = computed tomography, CTO = chronic total occlusion, PCI = percutaneous coronary intervention

**Table 2:** Comparison of lesion and procedural characteristics between the true-sub-true and the PCI unsuccessful groups.

Variables	All (n = 43)	True-sub-true group (n = 16)	Unsuccessful group (n = 27)	P-value
Antegrade flow				
Entry abrupt type, n (%)	36 (83.7)	11 (68.8)	25 (92.6)	0.055
Entry bifurcation with abrupt, n (%)	21 (48.8)	6 (37.5)	15 (55.6)	0.204
Mid-island, n (%)	14 (23.6)	6 (37.5)	8 (29.6)	0.419
Collateral flow				
Rentrop classification				0.411
0, n (%)	1 (2.3)	0	1 (3.7)	
1, n (%)	3 (7.0)	0	3 (11.1)	
2, n (%)	14 (32.6)	5 (31.3)	9 (33.3)	
3, n (%)	25 (58.1)	11 (68.8)	14 (51.9)	
Exit abrupt type, n (%)	29 (67.4)	13 (81.3)	16 (59.3)	0.124
Exit bifurcation with abrupt, n (%)	19 (44.2)	12 (75.0)	7 (25.9)	0.002
Exit bifurcation angle				0.144
0–60 degree, n (%)	12/19 (63.2)	6/12 (50.0)	6/7 (85.7)	
60–120 degree, n (%)	7/19 (36.8)	6/12 (50.0)	1/7 (14.3)	
120–180 degree, n (%)	0/19	0/19	0/19	
Bridge type, n (%)	11 (25.6)	2 (12.5)	9 (33.3)	0.124
PCI site				0.001
RCA, n (%)	21 (48.8)	3 (18.8)	18 (66.7)	
LAD, n (%)	17 (39.5)	8 (50.0)	9 (33.3)	
LCX, n (%)	5 (11.6)	5 (31.3)	0	
Calcification, n (%)	16 (37.2)	2 (12.5)	14 (51.9)	0.010
Bend>45, n (%)	18 (41.9)	5 (31.3)	13 (48.1)	0.223
Occlusion length>20mm, n (%)	30 (69.8)	12 (75.0)	18 (66.7)	0.413
Re-try, n (%)	3 (7.0)	0	3 (11.1)	0.237
J-CTO score±SD	2.40±1.16	1.94±0.85	2.67±1.24	0.082
Quantitative coronary angiogram				
Reference diameter±SD (mm)	1.78±0.71	1.75±0.53	1.80±0.81	0.820
Lesion length±SD (mm)	28.5±13.8	28.8±10.2	28.4±15.8	0.490
Peri-procedural myocardial infarction, n (%)	1 (2.3)	1 (6.3)	0	0.372
Approach				0.390
Femoral artery, n (%)	5 (11.6)	2 (12.5)	3 (11.1)	
Radial artery, n (%)	1 (2.3)	1 (6.3)	0	
Bi-femoral artery, n (%)	16 (37.2)	4 (25.0)	12 (44.4)	
Femoral artery & radial artery, n (%)	21 (48.8)	9 (56.3)	12 (44.4)	
Volume of contrast media±SD (ml)	202.1±73.1	207.6±84.2	198.8±67.1	0.451
Total fluoroscopic time±SD (min)	84.0±31.0	67.8±31.4	93.7±26.9	0.006
Procedural time±SD (min)	174.2±50.8	167.4±63.6	178.8±42.4	0.511
Stent number±SD		1.5±0.9	0	<0.001
Total stent length±SD (mm)		53.4±30.6	0	<0.001-
Wire number±SD	5.5±2.1	5.4±2.2	5.5±2.0	0.832
Cross wire*				0.653
Sion series–, n (%)	1 (2.3)	1 (6.3)	0	
X-treme series, n (%)	1 (4.7)	1 (6.3)	1 (3.7)	
Ultimate bross 3, n (%)	1 (2.3)	0	1 (3.7)	
Gaia series, n (%)	17 (39.5)	6 (37.5)	11 (40.7)	
Conquest series, n (%)	22 (51.2)	8 (50.0)	14 (51.9)	
Micro-catheter				
Corsair, n (%)	39 (90.7)	14 (87.5)	25 (92.6)	0.479
Dual lumen catheter, n (%)	29 (67.4)	13 (81.3)	16 (59.3)	0.124
Other, n (%)	12 (27.9)	6 (37.5)	6 (22.2)	0.232
Collateral flow enhancement system**, n (%)	33 (76.7)	10 (62.5)	23 (85.2)	0.093
Anchor balloon technique, n (%)	2 (4.7)	0	2 (7.4)	0.389
Attempted procedure				
Standard antegrade penetration, n (%)	44 (100)	16 (100)	28 (100)	-
Parallel wire, n (%)	25 (58.1)	11 (68.8)	14 (51.9)	0.223
Retrograde channel track, n (%)	10 (23.3)	1 (6.3)	9 (33.3)	0.044
IVUS guide wire, n (%)	6 (14.0)	4 (25.0)	2 (7.4)	0.125
Final procedure				
Standard antegrade penetration, n (%)		5 (31.3)		
Parallel wire, n (%)		7 (43.8)		
IVUS guide wire, n (%)		4 (25.0)		

Data are expressed as the mean±SD or number (percentage). A Student’s *t* test was used for normally distributed continuous variables, a Mann-Whitney *U* test was used for abnormally distributed continuous variables, and a Fisher exact test was used for categorical variables.

RCA = right coronary artery, LAD = left anterior descending artery, LCX = left circumflex artery.

* The heaviest tip load wire in unsucess cases

**Including retrograde penetration, only retrograde enhancement, and selective injection using micro-catheter from other branch

The univariate and multivariate logistic regression analysis is shown in Table 3. The model included the following variables with *p*<0.05 in univariate logistic regression analysis. Non-RCA (p = 0.004), bifurcation with, abrupt type (p = 0.018), and calcification (p = 0.017) were included in the model. The multivariate regression analysis revealed that bifurcation with abrupt type in CTO exit-site (OR 8.017; 95%CI: 1.484–43.304; p = 0.016) was independent predictor of the antegrade true-sub-true phenomenon.

**Table 3:** Determinants of antegrade true-sub-true: Univariate and multivariate logistic regression analysis.

Dependent variable: antegrade true-sub-true						
	Univariate Logistic Regression Analysis			Multivariate Logistic Regression Analysis		
	OR	95% CI	P value	OR	95% CI	P value
*Independent variables*						
Age	0.996	0.940–1.054	0.996			
Female (vs male)	-	-	-			
BSA (0.1 increase)	1.178	0.849–1.634	0.328			
Hypertension	-	-	-			
Hyperlipidemia	-	-	-			
Diabetes mellitus	0.392	0.089–1.721	0.392			
Chronic kidney disease	0.646	0.183–2.284	0.498			
CT angiography before CTO-PCI	1.100	0.292–4.142	0.888			
Non-RCA (vs RCA)	8.667	1.956–38.405	0.004	4.446	0.802–24.655	0.088
Entry abrupt type	0.176	0.029–1.051	0.057			
Entry bifurcation with abrupt type	0.480	0.135–1.710	0.480			
Mid-island	1.425	0.386–5.262	0.595			
Distal abrupt type	2.979	0.684–12.976	0.146			
Distal bifurcation with abrupt type	8.571	2.068–35.523	0.003	8.017	1.484–43.304	0.016
Bridge type	0.286	0.053–1.539	0.145			
Calcification	0.133	0.025–0.700	0.017	0.151	0.021–1.066	0.058
Bend>45	2.043	0.557–7.488	0.281			
Occlusion>20mm	1.500	0.375–5.998	0.556			
Re-try	-	-	-			

Univariate logistic regression analysis was performed to identify variables that had marginal association with antegreade true-sub-true (P < 0.05).

OR = odds ratio; CI = confidence interval; BSA = body surface area; CTO = chronic total occlusion; PCI = percutaneous coronary intervention

## Discussion

In the present study, we compared the clinical, lesion, and procedural characteristics between the true-sub-true group (n = 16) and the unsuccessful group (n = 27) to investigate specific conditions or characteristics that were associated with “antegrade true-sub-true” phenomenon in CTO-PCI. Multivariate analysis revealed that distal bifurcation with abrupt type was significantly associated with the “antegrade true-sub-true” phenomenon. Although the antegrade true-sub-true phenomenon has been considered to be a rare and accidental condition, it can be a last chance to success when all techniques including reverse CART and IVUS guide wiring would not work. Our results may shed light on the mechanism of antegrade true-sub-true phenomenon.

**Fig 4:**
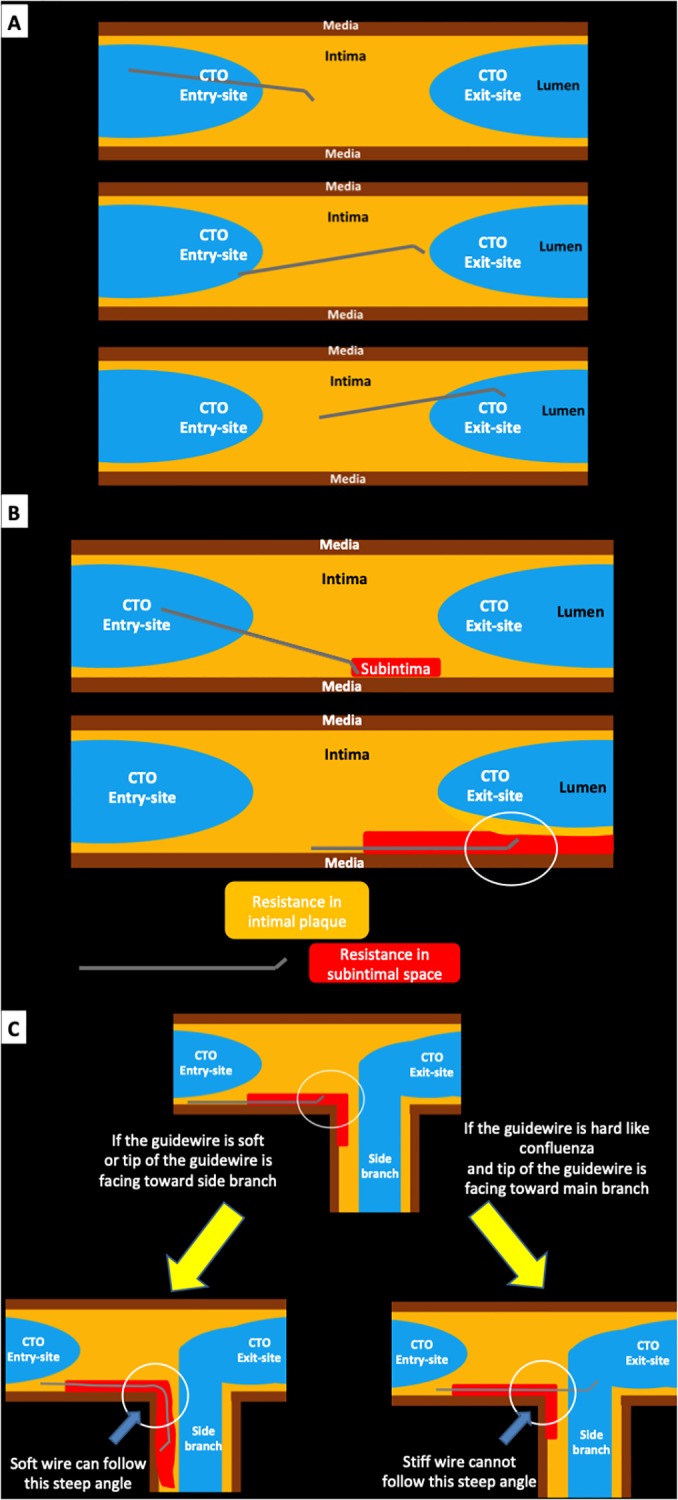
The schema of “antegrade true-sub-true” phenomenon. Panel A: A scheme describing conventional antegrade wiring. The guidewire advances from CTO entry-site to exit-site through intraplaque (intima). Panel B: If the guidewire get into the subintimal space, it is difficult to advance the guidewire from the subintimal space to the true lumen at CTO exit site, because the resistance in intimal plaque is greater than that in subintimal space. Panel C: The schema of the antegrade “true-sub-true” phenomena.

The presence of bifurcation with abrupt type in CTO exit-site was significantly associated with the “antegrade true-sub-true” phenomenon, which has not been sufficiently discussed in literatures. We would like to discuss why the “antegrade true-sub-true” phenomenon happen under the presence of bifurcation with abrupt type in CTO exit-site using original schemes. In the antegrade wire based approach, operators should try to advance the guidewire from CTO entry-site to exit-site through intraplaque (intima) (Fig 4A). However, if the guidewire get into the subintimal space, it is difficult to advance the guidewire from the subintimal space to the lumen in CTO exit-site, because the resistance in intimal plaque is greater than that in subintimal space (Fig 4B) [18, 19]. Therefore, if IVUS shows that the guidewire is within the subintimal space, operators try to pullback the guidewire until the intraplaque (intima) in IVUS-guided wiring [15]. On the other hand, if the configuration of CTO exit-site is bifurcation with abrupt type, the chance of the sub-true phenomenon may increase. In the setting of the guidewire within the subintimal space near the CTO exit-site (upper panel in Fig 4C), if the guidewire is soft or the tip of the guidewire is facing toward the side branch, the guidewire would advance into the subintimal space of the side branch, because the soft guidewire can follow the steep angle (lower left panel in Fig 4C). If the guidewire is hard like Confianza (conquest) family and the tip of the guidewire is facing toward the main branch, the guidewire would advance into the true lumen in CTO exit-site, because the stiff wire cannot follow the steep angel (lower right panel in Fig 4C). Of course, we have to admit that the above explanation is a speculation, but it came from our experience in CTO pathology [18].

Our results also revealed that the non-RCA-CTO tended to be associated with the “antegrade true-sub-true” phenomenon as compared to the RCA-CTO. In general, the RCA has less side branches as compared to the LAD or LCX. Therefore, the chance of bifurcation with abrupt type in CTO exit-site, which was associated with the antegrade true-sub-true phenomenon, might be less in the RCA-CTO. On the other hand, the presence of calcification tended to be associated with unsuccessful CTO-PCI. Since the strong association between calcification and unsuccessful CTO-PCI has often reported in literatures [5, 29, 30], it was not surprising that calcification was inversely associated with the true-sub-true phenomenon. Our speculation regarding successful wiring (Fig 4) would not work under the severe calcification, especially calcification near the CTO exit-site.

**Fig 5:**
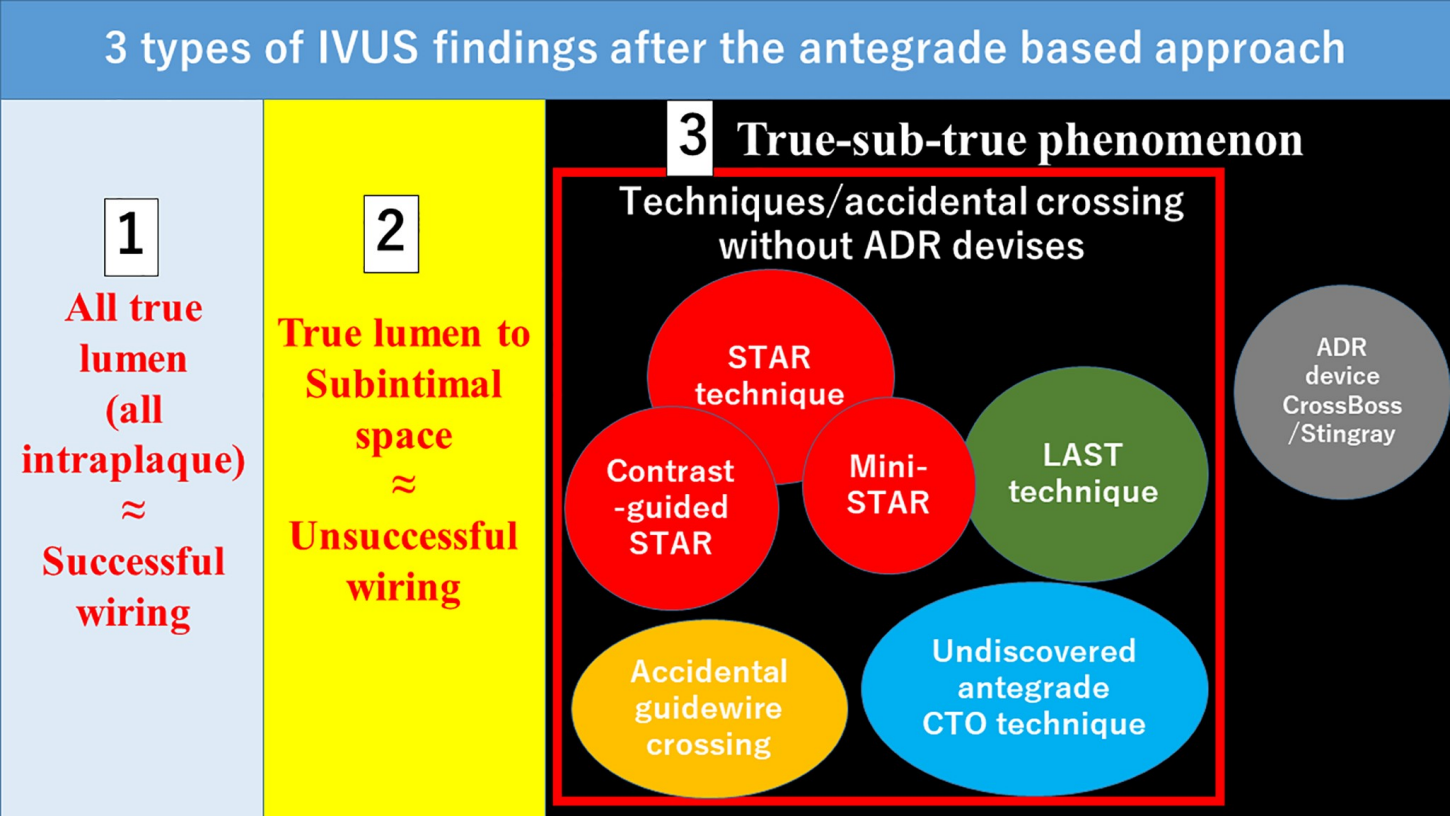
The scheme of 3 types of IVUS findings after the antegrade based approach.

Subintimal tracking and reentry (STAR) technique was firstly reported by Colombo, et al. in 2005 [12]. Contrast-guided STAR and mini-STAR techniques were developed as a modification of STAR technique [31, 32]. Moreover, limited antegrade subintimal tracking (LAST) technique was also reported as an option of wire-based dissection re-entry [33]. Although IVUS is not a requisite for STAR technique, min-STAR technique, LAST technique, or CrossBoss/Stingray system, the antegrade true-sub-true phenomenon would be probably observed in those techniques if IVUS would be performed after the guidewire cross. In the antegrade approach, the final IVUS findings would be categorized into 3 types: (1) all true lumen (successful wiring), (2) true-subintima (unsuccessful wiring), or (3) true-sub-true phenomenon (Fig 5). Therefore, the concept of the antegrade true-sub-true phenomenon would include STAR technique, mini-STAR technique, contrast-guides STAR technique, LAST technique, and CrossBoss/Stingray system. Accidental (unintentional) guidewire crossing, which was the main reason of true-sub-true phenomenon in the present study, would be included in the true-sub-true phenomenon. Moreover, undiscovered antegrade CTO techniques may be included in the true-sub-true phenomenon. The recent CTO algorithm did not include STAR technique as an option [11]. However, wire-based dissection re-entry techniques including STAR technique may be used as a last option in the current CTO-PCI. Our finding may provide an additional insight into the key to success in wire-based dissection re-entry techniques.

Clinical implications of the present study should be noted. First, if the configuration of CTO exit-site is bifurcation with abrupt type, operators can recognize that there is a chance for antegrade true-sub-true crossing. However, even if there is a chance for antegrade true-sub-true crossing, it would not be easy to advance the guidewire within the subintimal space into the true lumen intentionally. In fact, the IVUS guide wiring was attempted in 4 CTO-lesions in the true-sub-true group. In those 4 lesions, we tried to advance the guidewire through intraplaque using IVUS guide, but we could not. Thus, the successful true-sub-true wiring did not come from the intentional tracking. Therefore, the antegrade true-sub-true wiring should be the last resort after all options such as parallel wiring or retrograde approach would not work. In other words, if the configuration of CTO exit-site is not bifurcation with abrupt type, it would be practical to abandon antegrade true-sub-true wiring for safer CTO-PCI. To the best of our knowledge, this is the first study that investigated the determinants of “antegrade true-sub-true” phenomenon. Our study revealed that the antegrade true-sub-true phenomenon could happen under some conditions such as bifurcation with abrupt type in CTO exit-sites.

### Study limitations

First, since this study was a retrospective observational study, there is a risk of patient selection bias and group selection bias. Second, the success rate of CTO-PCI as well as the complications of CTO-PCI would highly depend on the operators. This single center study may not reflect the contemporary procedures for CTO-PCI, while the success rate of CTO-PCI during the study period seems to be equal or greater than contemporary CTO-PCI registries [5, 6, 24]. Third, because we could not perform IVUS by auto-pullback mode, we could not perform longitudinal analysis such as subintimal lesion and location of distal bifurcation. Forth, because IVUS findings were evaluated after pre-dilatation following the wire crossing, we could not exclude the possibility that a part of guidewire moved from the intimal space to the subintimal space after balloon dilatation, especially when the guidewire advanced near the subintimal space. In other words, the true-sub-true group might include lesions in which the guidewire advanced through intraplaque from CTO entry-site to exit-site. That might explain that several cases using soft wire were included in antegrade true-sub-true group. Fifth, the coronary angiograms were not revised by independent operators. Sixth, we did not include lesions that were crossed by standard antegrade penetration through intraplaque as the competitor with antegrade true-sub-true group, because antegrade true-sub-true phenomenon would probably happen after the unsuccessful standard antegrade penetration. Seventh although the concept of the antegrade true-sub-true phenomenon was different from STAR technique, the phenomenon has a potential to be the result of STAR technique whether procedure was performed intentionally or not. Eighth, our study could not include the cases treated with antegrade dissection re-entry (ADR) devises, because the ADR device was only available in limited facilities during the study period in Japan. On the other hand, there were many chances to use parallel wire techniques instead of ADR devises. These situations (no ADR devices, but frequent parallel wire techniques) would limit the definition of contemporary CTO-PCI techniques in this manuscript. Then Finally, we should mention the difference between our antegrade true-sub-true phenomenon and ADR. Unlike our antegrade true-sub-true phenomenon, ADR does not require a side branch at the re-entry point. Moreover, the success rate of ADR by experienced operators were approximately 90% [34]. Therefore, our antegrade true-sub-true phenomenon cannot be an alternative to ADR.

## Conclusions

In CTO-PCI, the antegrade true-sub-true phenomenon is rare, but can be a last chance for successful PCI. Bifurcation with abrupt type in CTO exit-site was significantly associated with the antegrade true-sub-true phenomenon. Our results suggest that the antegrade true-sub-true phenomenon can happen under specific pathohistological conditions rather than accidentally.

## Supporting information

S1 Data (XLSX)
